# Exercise Improves Sarcopenic Obesity Through Inhibition of Ferroptosis and Activation of the AMPK/ACC Pathway

**DOI:** 10.3390/ijms27031187

**Published:** 2026-01-24

**Authors:** Qin Ru, Congyue Xu, Chongzhou Wan, Bei Cheng, Xiao Xiang, Li Fang, Junqing Ren, Lin Chen, Yuxiang Wu

**Affiliations:** Institute of Intelligent Sport and Proactive Health, Department of Health and Physical Education, Jianghan University, Wuhan 430056, China; ruqin@jhun.edu.cn (Q.R.); xucy@jhun.edu.cn (C.X.); 18653228830@163.com (C.W.); chengbei08@163.com (B.C.); m13403047529@163.com (X.X.); 15549223507@163.com (L.F.); k256654@126.com (J.R.); lchen@jhun.edu.cn (L.C.)

**Keywords:** sarcopenic obesity, exercise intervention, ferroptosis, AMPK/ACC pathway, iron-lipid metabolism

## Abstract

Sarcopenic obesity, characterized by skeletal muscle loss concurrent with adipose tissue accumulation, has emerged as a global health threat. Exercise is established as an effective intervention; however, the molecular mechanisms underlying its protective effects remain incompletely defined. This study investigated whether exercise mitigates high-fat diet (HFD)-induced sarcopenic obesity, and whether the mechanism was related to the activation of the adenosine monophosphate-activated protein kinase (AMPK)/Acetyl-CoA carboxylase pathway (ACC) pathway and the inhibition of ferroptosis. Cell experiments demonstrated that palmitic acid induced ferroptosis in C2C12 mouse myoblasts. Animal experiments confirmed that HFD promoted skeletal muscle ferroptosis in C57BL/6 mice, evidenced by iron metabolism imbalance (solute carrier family 39 member14 upregulation, ferroportin downregulation), impaired antioxidant capacity (reduced glutathione, superoxide dismutase, glutathione peroxidase 4), and elevated lipid peroxidation (increased malondialdehyde). Meanwhile, both flat treadmill running and uphill treadmill running may reverse these changes by activating AMPK/ACC phosphorylation, reducing non-transferrin iron uptake, enhancing iron export and storage, and improving antioxidant status, jointly inhibiting ferroptosis and attenuating muscle mass loss and lipid deposition. These findings confirm that ferroptosis acts as one of the key pathogenic drivers in sarcopenic obesity and suggests that exercise may improve sarcopenic obesity by activating the AMPK/ACC pathway and inhibiting ferroptosis. This study provides novel mechanistic insights into exercise-mediated regulation of iron-lipid metabolism crosstalk and informs targeted interventions for sarcopenic obesity.

## 1. Introduction

Overweight and obesity have escalated into a global epidemic [1,2]. Over the past three decades, global age-standardized disability-adjusted life years (DALYs) related to overweight/obesity have increased by more than 15%, ranking it among the fastest-growing and leading attributable health burdens [3]. It is worth noting that obesity can induce skeletal muscle loss and functional decline through hormonal imbalances, chronic inflammation, insulin resistance, oxidative stress, and metabolic disturbances, thereby contributing to the development of sarcopenia [4,5,6,7,8,9]. Sarcopenic obesity, as defined by the European Society for Clinical Nutrition and Metabolism (ESPEN)/European Association for the Study of Obesity (EASO) 2022 consensus statement [10], is a syndrome characterized by concurrent loss of skeletal muscle mass/function and excessive adipose tissue accumulation. This condition confers higher risks of metabolic disorders, physical disability, and mortality compared to obesity or sarcopenia alone [11], emerging as a critical global public health concern [12,13]. The maladaptive interaction between adipose tissue expansion and skeletal muscle dysfunction, coupled with the complex crosstalk between lipid metabolism dysregulation and muscle atrophy, complicates therapeutic development for this condition [14,15], necessitating novel mechanistic insights to guide precision interventions.

Exercise is a well-established effective intervention for sarcopenic obesity [16,17], and multiple meta-analyses have demonstrated that moderate physical exercise increases muscle strength and muscle mass while decreasing fat mass [18,19,20]. Despite these well-documented benefits, the molecular pathways underlying exercise-induced protective effects remain not fully elucidated. Previous studies have primarily focused on inflammation regulation, mitochondrial biogenesis, and oxidative stress regulation [21,22,23,24]. However, recent research suggests that exercise may ameliorate sarcopenic obesity by modulating ferroptosis.

Ferroptosis is characterized by iron-dependent, irreversible accumulation of lipid peroxides beyond the cellular antioxidant capacity, primarily regulated by glutathione peroxidase 4 (GPX4) and iron metabolism pathways [25]. Emerging evidence implicates ferroptosis in the pathogenesis of both muscle wasting and adipose dysfunction [26]. In skeletal muscle, iron dyshomeostasis, characterized by increased labile iron accumulation and impaired iron sequestration, promotes ferroptosis in myocytes, contributing to muscle mass reduction and impaired muscle function [27]. It is crucial to note that ferroptosis in skeletal muscle has a unique tissue-specific regulatory mechanism. Myokines secreted by contracting skeletal muscle, such as irisin, enhance antioxidant capacity by activating the GPX4/ solute carrier family 7 member 11 (SLC7A11) pathway [28,29]; mitophagy is crucial for mitochondrial quality control in muscle cells, as it regulates intracellular iron homeostasis by clearing iron-loaded damaged mitochondria [30], and the high mitochondrial density in skeletal muscle inherently increases the sensitivity of muscle cells to iron overload-induced lipid peroxidation [31]. Low levels of ferroptosis markers are present in the adipose tissues of obese individuals and obese mice, correlating with adipose inflammation and insulin resistance [32]. Activating ferroptosis signaling in adipose tissue leads to the degradation of hypoxia-inducible factor-1 α, thereby relieving the inhibition of the thermogenic program regulated by the peroxisome proliferator-activated receptor γ coactivator-1β (Pgc-1β) pathway, which may help prevent and treat obesity and its related metabolic disorders [33]. However, the specific role of ferroptosis in sarcopenic obesity remains unexplored.

Notably, adenosine monophosphate-activated protein kinase (AMPK), a central regulator of cellular energy homeostasis [34], has been implicated in both lipid metabolism and cell survival. Upon activation, AMPK phosphorylates and inhibits its downstream substrate acetyl-CoA carboxylase (ACC), thereby enhancing fatty acid oxidation and reducing lipid accumulation [35,36]. Recent studies have further revealed that the AMPK/ACC pathway regulates lipid metabolism homeostasis and mitigates lipid peroxidation during ferroptosis, with AMPK activation under energy stress emerging as a potential regulatory mechanism for ferroptosis [37,38]. Given the central role of the AMPK/ACC pathway in energy regulation and the metabolic sensitivity of ferroptosis [39,40], it is hypothesized that exercise-induced activation of the AMPK/ACC pathway may suppress muscle ferroptosis, thereby contributing to the improvement of sarcopenic obesity [41]. Therefore, this study aimed to evaluate the effect of exercise intervention on high-fat diet (HFD)-induced sarcopenic obesity in mice. Furthermore, by analyzing the interplay among exercise, ferroptosis, and the AMPK/ACC pathway, we sought to elucidate the specific mechanism by which exercise improves sarcopenic obesity by regulating the AMPK/ACC pathway and inhibiting ferroptosis in skeletal muscle.

## 2. Results

### 2.1. Ferroptosis Contributes to Palmitic Acid-Induced Inhibition of Proliferation and Myogenic Differentiation in C2C12 Cells

**Figure 1:**
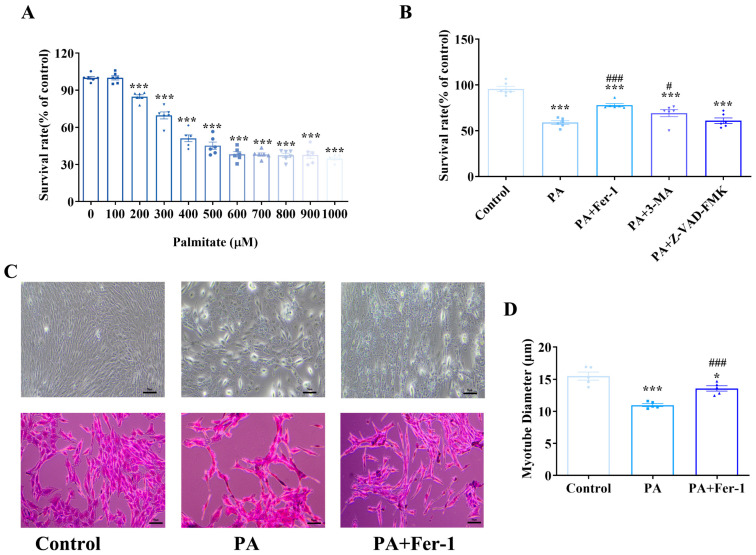
Ferroptosis contributes to palmitic acid-induced inhibition of proliferation and myogenic differentiation in C2C12 cells. (**A**) Palmitic acid (PA) inhibits the proliferation of C2C12 cells; MTT (3-(4,5-dimethylthiazol-2-yl)-2,5-diphenyltetrazolium bromide) assays were conducted at least three times, with representative results shown; (**B**) The effects of different inhibitors on the cytotoxicity of C2C12 cells induced by PA (500 μmol/L) and different cell death inhibitors, including ferroptosis inhibitor ferrostatin-1 (Fer-1, 10 μmol/L), autophagy inhibitor 3-methyladenine (3-MA,1 mmol/L), and apoptosis inhibitor z-VAD-FMK (25 μmol/L), were combined with PA to treat C2C12 cells for 24 h; MTT assays were conducted at least three times, with representative results shown; (**C**) Morphological detection of myogenic differentiation of C2C12 cells, scale bar = 50 μm; (**D**) PA can inhibit the myogenic differentiation of C2C12 cells, which can be reversed by the ferroptosis inhibitor Fer-1 (*n* = 5). * Compared with the control group, *p* < 0.05, *** compared with the control group, *p* < 0.001; # compared with the PA group, *p* < 0.05, ### compared with the PA group, *p* < 0.001.

An in vitro high-fat-induced myoblast injury model was established using palmitic acid (PA). The MTT (3-(4,5-dimethylthiazol-2-yl)-2,5-diphenyltetrazolium bromide)-based cell viability assay demonstrated that exposure of C2C12 myoblasts to graded concentrations of PA resulted in significant reduction in cellular proliferation and cell viability (Figure 1A). To elucidate the potential molecular mechanism underpinning PA-induced cytotoxicity, a panel of pharmacologic inhibitors targeting distinct cell death pathways, including ferroptosis inhibitor ferrostatin-1 (Fer-1), autophagy inhibitor 3-methyladenine (3-MA), and pan-caspase inhibitor Z-VAD-FMK, was employed (Figure 1B). Notably, 3-MA treatment significantly mitigated PA-induced cytotoxicity, as evidenced by a statistically significant increase in cell viability (*p* < 0.05), suggesting a contributory role of autophagic dysregulation in PA-mediated cellular stress. However, Fer-1 emerged as the most efficacious intervention to alleviate PA-induced cytotoxicity, demonstrating a marked restoration of cell viability with a highly significant statistical difference (*p* < 0.001), although Fer-1 treatment alone had no significant effect on cell viability (Figure S1). These findings strongly suggest that PA primarily reduces cell viability by promoting ferroptosis, highlighting the crucial role of oxidative stress and iron-dependent lipid peroxidation in PA-induced cell damage.

Myogenic differentiation of C2C12 cells was assessed via conventional light microscopy and hematoxylin-eosin (HE) staining (Figure 1C). In the control group, C2C12 cells underwent normal myogenic differentiation, characterized by the formation of slender, multinucleated myotubes with a typical fusiform morphology. Quantitative analysis showed that the average diameter of the myotubes was 15.51 ± 1.20 μm (Figure 1D). In contrast, PA impaired myogenic differentiation of C2C12 cells, with a reduction in the number of differentiated myotubes. Measurements of myotube diameter revealed a significant decrease, with an average of 10.97 ± 0.26 μm (*p* < 0.001 compared with the control group), indicating that PA exposure not only inhibited myoblast differentiation but also led to thinner myotubes. Notably, Fer-1 intervention improved myogenic differentiation, with the formation of slender myotubes whose morphology resembled that of the control group. Quantitative analysis confirmed a significant increase in myotube diameter in the Fer-1-treated group compared with the PA-induced model group (*p* < 0.001). These results suggest that Fer-1 may effectively rescue PA-induced inhibition of myogenic differentiation in C2C12 cells, by regulating ferroptosis-related pathways, and promote the growth and maturation of myotubes by restoring myotube diameters.

### 2.2. Palmitic Acid Exposure Leads to Iron Overload and Lipid Peroxidation in C2C12 Cells

**Figure 2:**
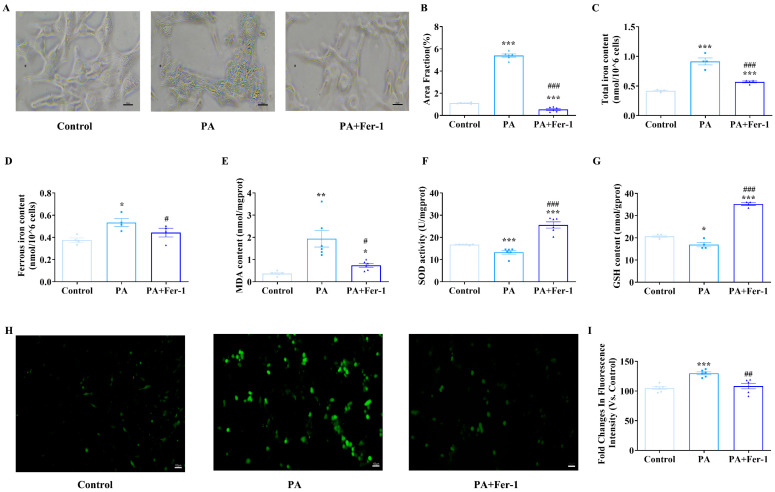
Palmitic acid (PA) exposure leads to iron overload and lipid peroxidation in C2C12 cells. (**A**) Iron staining of C2C12 cells, scale bar = 20 μm; (**B**) statistical results of iron staining of C2C12 cells (*n* = 5); (**C**) detection of total iron content in cells of each group (*n* = 4); (**D**) detection of ferrous iron content in cells of each group (*n* = 4); (**E**) PA treatment (500 μmol/L) increases malondialdehyde (MDA) content in C2C12 cells, which can be reversed by ferroptosis inhibitor Fer-1 (10 μmol/L, *n* = 6); (**F**) PA treatment reduces superoxide dismutase (SOD) activity in C2C12 cells, which can be reversed by ferroptosis inhibitor Fer-1 (*n* = 4); (**G**) PA treatment decreases glutathione (GSH) content in C2C12 cells, which can be reversed by ferroptosis inhibitor Fer-1 (*n* = 4); (**H**) fluorescence microscopic morphological detection of reactive oxygen species (ROS) content, scale bar = 100 μm; (**I**) PA treatment promotes ROS production in C2C12 cells, which can be reversed by ferroptosis inhibitor Fer-1 (*n* = 6). * Compared with the control group, *p* < 0.05, ** compared with the control group, *p* < 0.01, *** compared with the control group, *p* < 0.001; # compared with the PA group, *p* < 0.05, ## compared with the PA group, *p* < 0.01, ### compared with the PA group, *p* < 0.001.

Key hallmarks of ferroptosis include iron accumulation, lipid peroxidation and glutathione (GSH) depletion [42,43]. Iron staining results showed that compared with the control group, PA-treated cells exhibited significantly increased iron staining intensity, evidenced by deeper coloration and more abundant iron-positive granules (Figure 2A,B, *p* < 0.001). In contrast, Fer-1 treatment effectively reversed PA-induced iron accumulation. Consistent findings were obtained in measurements of cellular total iron and ferrous iron content (Figure 2C,D). These findings indicate that PA promotes intracellular iron deposition, while Fer-1 can counteract this effect, suggesting potential involvement of ferroptosis-related pathways in PA-induced iron metabolism dysregulation.

Subsequently, the antioxidant capacity and lipid peroxidation of C2C12 cells after PA treatment were detected. Compared with the control group, the PA-treated group showed significantly elevated malondialdehyde (MDA) levels, which were effectively attenuated by Fer-1 co-treatment (Figure 2E). PA exposure also led to pronounced reductions in superoxide dismutase (SOD) activity and GSH content compared to controls; however, Fer-1 administration significantly restored SOD activity and elevated GSH levels (Figure 2F,G). C2C12 cells treated with PA exhibited a significantly higher relative fluorescence intensity than controls, indicating increased reactive oxygen species (ROS) production (Figure 2H,I). Conversely, co-treatment with PA and Fer-1 resulted in significantly lower relative fluorescence intensity compared to PA alone. This finding strongly suggests that Fer-1 can effectively mitigate the PA-induced ROS elevation, likely through inhibiting ferroptosis. Collectively, these changes in oxidative stress-related indicators and their reversal by Fer-1 further confirm that PA inhibits myoblast proliferation by inducing iron-dependent lipid peroxidation, implicating ferroptosis-related mechanisms in PA-mediated myoblast dysfunction.

### 2.3. Palmitic Acid Causes Mitochondrial Morphological Changes in C2C12 Cells

**Figure 3:**
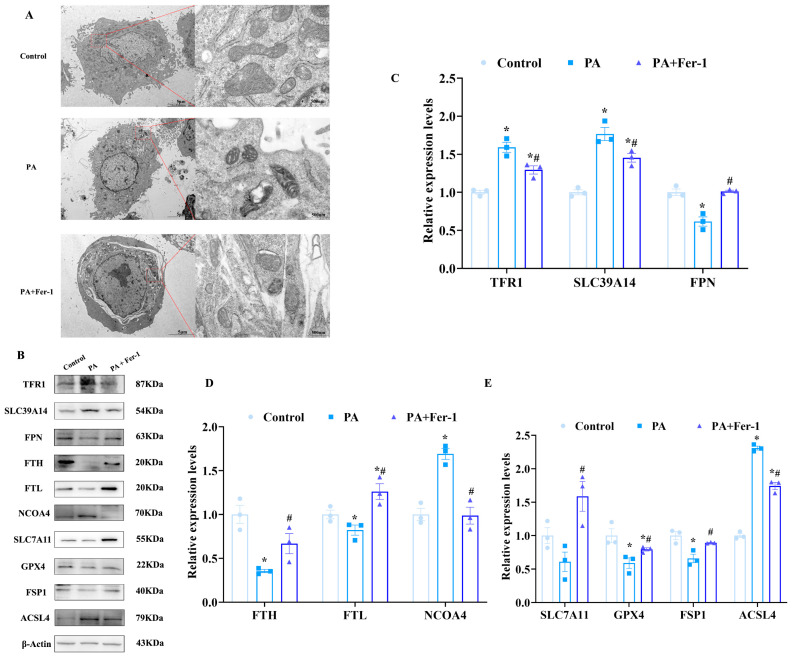
Palmitic acid causes ferroptosis in C2C12 cells. (**A**) PA (500 μmol/L) causes mitochondrial morphological changes in C2C12 cells, which can be reversed by ferroptosis inhibitor Fer-1 (10 μmol/L, *n* = 3), scale bar(left) = 5 μm, scale bar(right) = 500 nm.; (**B**) expression of ferroptosis-related proteins; (**C**) expression analysis of transferrin receptor 1 (TFR1), solute carrier family 39 member 14 (SLC39A14) and ferroportin (FPN) in C2C12 cells (*n* = 3); (**D**) expression analysis of ferritin heavy chain (FTH), ferritin light chain (FTL) and nuclear receptor coactivator 4 (NCOA4) in C2C12 cells (*n* = 3); (**E**) expression analysis of solute carrier family 7 member 11 (SLC7A11), glutathione peroxidase 4 (GPX4), ferroptosis suppressor protein 1 (FSP1), and Acyl-CoA synthetase long-chain family member 4 (ACSL4) in C2C12 cells (*n* = 3). * Compared with the control group, *p* < 0.05; # compared with the PA group, *p* < 0.05.

Previous studies have indicated unique mitochondrial morphological characteristics during ferroptosis [44,45]. To further verify PA-induced ferroptosis in C2C12 cells, we examined mitochondrial ultrastructure using a transmission electron microscope (TEM). As shown in Figure 3A, C2C12 cells treated with PA exhibited distinct mitochondrial morphological changes compared to controls. Specifically, the mitochondria in PA-treated cells exhibited significantly increased membrane density and remarkably reduced cristae structures, which are consistent with the morphological features of mitochondria during ferroptosis reported previously [46]. In contrast, the abnormal mitochondrial morphology was largely reversed after Fer-1 intervention, manifested as reduced mitochondrial membrane density and restored cristae structures, suggesting that Fer-1 effectively alleviates PA-induced ferroptosis-related mitochondrial morphological changes in C2C12 cells.

### 2.4. Fer-1 Reverses Ferroptosis in C2C12 Cells Induced by Palmitic Acid

To further confirm PA-induced ferroptosis in C2C12 cells, Western blotting was performed to assess the expression of ferroptosis-related proteins. PA treatment significantly upregulated the expression of transferrin receptor 1 (TFR1), solute carrier family 39 member14 (SLC39A14), and nuclear receptor coactivator 4 (NCOA4) in C2C12 cells (Figure 3B,C). TFR1 and SLC39A14 are involved in iron uptake, while NCOA4 plays a crucial role in ferritinophagy. Upregulation of these proteins indicates increased intracellular free iron levels. Conversely, after PA treatment, the expression of ferritin light chain (FTL) and ferritin heavy chain (FTH) in C2C12 cells was downregulated (Figure 3D). As core components of ferritin, a key iron storage protein that sequesters iron in a non-toxic form, reduced FTL and FTH expression suggests diminished iron storage capacity, consequently resulting in an increase in free iron and facilitating the onset of ferroptosis.

Acyl-CoA synthase long-chain family member 4 (ACSL4) is a key enzyme in the ferroptosis pathway. The enhanced expression of ACSL4 in C2C12 cells subsequent to PA treatment further confirmed the inducing effect of PA on ferroptosis. Moreover, the expression of solute carrier family 7 member 11 (SLC7A11) and glutathione peroxidase 4 (GPX4) were downregulated following PA treatment (Figure 3E). SLC7A11 is responsible for the uptake of cystine, which is crucial for the synthesis of glutathione, and its downregulation impairs glutathione production to promote ferroptosis. GPX4 functions as a vital antioxidant enzyme that safeguards cells from lipid peroxidation and a decline in its expression exacerbates ferroptosis.

Notably, Fer-1 effectively inhibited PA-induced cell ferroptosis in C2C12 cells. Specifically, Fer-1 reversed PA-induced upregulation of TFR1, ACSL4, and NCOA4, significantly reducing their expression levels, while alleviating the PA-induced downregulation of SLC7A11, GPX4, FTL, and FTH. These results indicate that PA induces ferroptosis in C2C12 cells, while Fer-1 exerts a significant inhibitory effect by regulating multiple ferroptosis-related proteins.

### 2.5. Exercise Reduces HFD-Induced Increase in Body Weight and Body Fat Weight

**Figure 4:**
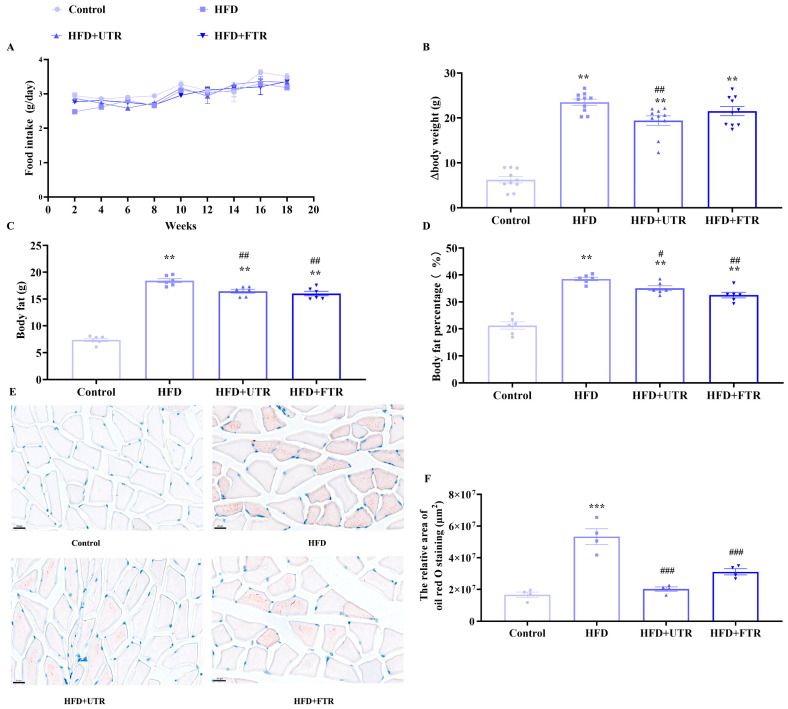
Exercise reduces lipid deposition in skeletal muscle induced by high-fat diet (HFD). (**A**) Changes in the food intake of mice in each group during the experiment; (**B**) changes in body weight gain of mice in each group during the experiment (*n* = 10); (**C**) exercise improves the increase in body fat in mice induced by HFD (*n* = 6); (**D**) exercise reduces the increase in body fat percentage in mice induced by HFD (*n* = 6); (**E**) gastrocnemius muscle oil red O staining of mice in each group, scale bar = 20 μm; (**F**) statistics of oil red O staining (*n* = 4). UTR: uphill treadmill running, FTR: flat treadmill running; ** Compared with the control group, *p* < 0.01, *** compared with the control group, *p* < 0.001; # compared with the HFD group, *p* < 0.05, ## compared with the HFD group, *p* < 0.01; ### compared with the HFD group, *p* < 0.001.

In animal experiments, a murine model of sarcopenic obesity was successfully established through a chronic high-fat diet (HFD), followed by interventions of flat or uphill treadmill running. Food intake did not differ significantly among groups throughout the experiment (Figure 4A). Mice in the model group exhibited a significantly greater increment in body weight compared to the control group (Figure 4B). Notably, both flat and uphill treadmill running effectively attenuated the HFD-induced body weight gain, as evidenced by statistically significant reductions in weight increment. Body fat mass and percentage were quantified using a small-animal body composition analyzer. Compared with the control group, the body fat weight and body fat percentage of mice in the HFD group increased significantly (Figure 4C,D). Both flat and uphill treadmill running interventions effectively reversed HFD-induced elevations in body fat mass and percentage.

### 2.6. Exercise Reduces Lipid Deposition in Skeletal Muscle Induced by HFD

Oil Red O staining of gastrocnemius muscle sections revealed minimal lipid droplets within muscle fibers in the control group (Figure 4E,F). In contrast, the HFD model group exhibited significant lipid deposition, with numerous lipid droplets distributed throughout muscle fibers, indicating abnormal lipid accumulation in the gastrocnemius muscle under HFD conditions. After exercise intervention, Oil Red O staining demonstrated a marked reduction in the number and size of lipid droplets in gastrocnemius muscle fibers. These findings indicate that both flat and uphill treadmill running can effectively improve the lipid metabolic dysfunction of skeletal muscle, thereby reducing lipid deposition.

### 2.7. Exercise Improves the Reduction in Skeletal Muscle Mass and Functional Decline Induced by HFD

Sarcopenia is characterized by reduced skeletal muscle mass and impaired function, and the effects of flat and uphill treadmill running on HFD-induced declines in skeletal muscle mass and function were investigated. Compared with the control group, skeletal muscle strength was significantly reduced in the HFD group, while both flat and uphill treadmill running could reverse the decline in skeletal muscle strength caused by HFD.

**Figure 5:**
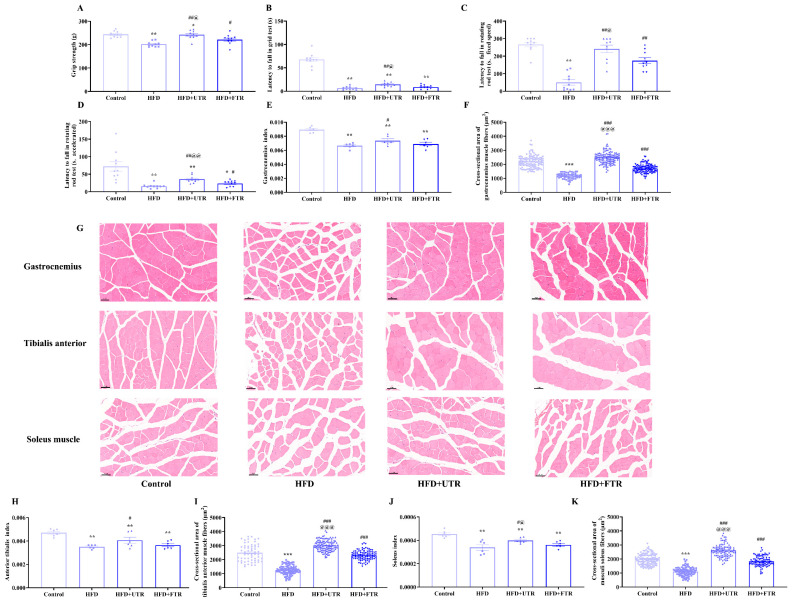
Exercise improves sarcopenia induced by high-fat diet (HFD). (**A**) Exercise improves the decline in grip strength induced by HFD in mice (*n* = 10); (**B**) exercise increases the latency to fall of mice in the grid test (*n* = 10); (**C**) exercise increases the latency to fall of mice in the rotating rod (fixed speed) (*n* = 10); (**D**) exercise increases the latency to fall of mice in the rotating rod (accelerated mode) (*n* = 10); (**E**) HFD induces a decrease in the gastrocnemius muscle index of mice, and exercise intervention increases the gastrocnemius muscle index of mice (*n* = 6); (**F**) exercise increases the cross-sectional area (CSA) of the gastrocnemius muscle fibers in mice (*n* = 3); (**G**) HE staining of the gastrocnemius muscle, tibialis anterior muscle, and soleus muscle of mice in each group, scale bar = 50 μm; (**H**) HFD induced a decrease in the tibialis anterior muscle index of mice, and exercise intervention increases the gastrocnemius muscle index of mice (*n* = 6); (**I**) exercise increases the CSA of the anterior tibial muscle fibers in mice (*n* = 3); (**J**) HFD induces a decrease in the soleus muscle index of mice, while exercise intervention increases the soleus muscle index of mice (*n* = 6); (**K**) exercise increases the CSA of soleus muscle fibers in mice (*n* = 3). UTR: uphill treadmill running, FTR: flat treadmill running. * Compared with the control group, *p* < 0.05, ** compared with the control group, *p* < 0.01, *** compared with the control group, *p* < 0.001; # compared with the HFD group, *p* < 0.05, ## compared with the HFD group, *p* < 0.01, ### compared with the HFD group, *p* < 0.001; @ compared with the flat treadmill running group, *p* < 0.05, @@ compared with the flat treadmill running group, *p* < 0.01, @@@ compared with the flat treadmill running group, *p* < 0.001.

In the grip strength test, the grip strength of mice in the HFD group was significantly lower than in controls; both flat and uphill treadmill running could enhance the grip strength of mice, with uphill treadmill running exerting a more pronounced effect than flat treadmill running (Figure 5A). The results of the hanging grid test indicated that compared with the control group, the latency to fall of mice in the HFD group was significantly shortened. Uphill treadmill running effectively increased latency to fall, suggesting a positive impact on maintaining muscle function and balance (Figure 5B). In the rotating rod test, compared with the control group, the latency to fall of mice in the HFD group was significantly reduced in both the fixed speed and accelerated rotation tests (Figure 5C,D). Both flat and uphill treadmill running increased latency to fall in these tests, with uphill treadmill running demonstrating significantly greater efficacy than flat treadmill running. Collectively, these results suggest that HFD induces sarcopenic obesity in mice, while both flat and uphill treadmill running ameliorate sarcopenic obesity to a certain extent, with uphill treadmill running showing a more prominent effect on improving skeletal muscle function.

We have also investigated the effect of flat and uphill treadmill running in improving skeletal muscle mass. Compared to controls, the gastrocnemius index, soleus muscle index, and tibialis anterior index of mice in the HFD group were significantly decreased (Figure 5E,H,J). Although flat treadmill running intervention trended toward increasing the skeletal muscle indices in mice, no significant differences were observed relative to the HFD group. In contrast, uphill treadmill running significantly reversed the decrease in skeletal muscle indices caused by the HFD, with the gastrocnemius muscle index, the soleus muscle index, and the tibialis anterior muscle index of mice in the uphill treadmill running group all significantly higher than those in the HFD group. These results indicate that HFD induces skeletal muscle mass loss in mice and uphill treadmill running may be superior to flat treadmill running in ameliorating muscle atrophy.

### 2.8. Exercise Increases the Cross-Sectional Area of Muscle Fibers

HE staining of the gastrocnemius muscle revealed that the muscle fibers were arranged in a compact and regular manner in the control group. The cytoplasm of the muscle fibers showed strong eosinophilia, being uniformly stained red. The nuclei, which were oval-shaped and multiple in number, were located at the periphery of the muscle fibers. In contrast, the HFD-induced model mice exhibited muscle fiber atrophy, characterized by uneven fiber thickness and scattered irregular cavity-like structures (Figure 5G). After flat treadmill running and uphill treadmill running interventions, gastrocnemius muscle structure was significantly improved, with more organized fiber arrangement and reduced atrophy.

Quantitative analysis of gastrocnemius muscle fiber cross-sectional area (CSA) showed a significant reduction in the HFD group compared to controls (Figure 5F). However, both flat and uphill treadmill running were capable of increasing the CSA of gastrocnemius muscle fibers, thereby ameliorating HFD-induced skeletal muscle atrophy. Consistent findings were observed in HE-stained sections of the soleus and tibialis anterior muscles (Figure 5I,K). These results suggest that HFD reduces skeletal muscle fiber CSA, while flat and uphill treadmill running effectively counteract this adverse effect, further supporting the role of exercise in improving muscle mass and preventing HFD-induced muscle atrophy.

### 2.9. Exercise Improves HFD-Induced Sarcopenic Obesity in Mice by Inhibiting Ferroptosis

**Figure 6:**
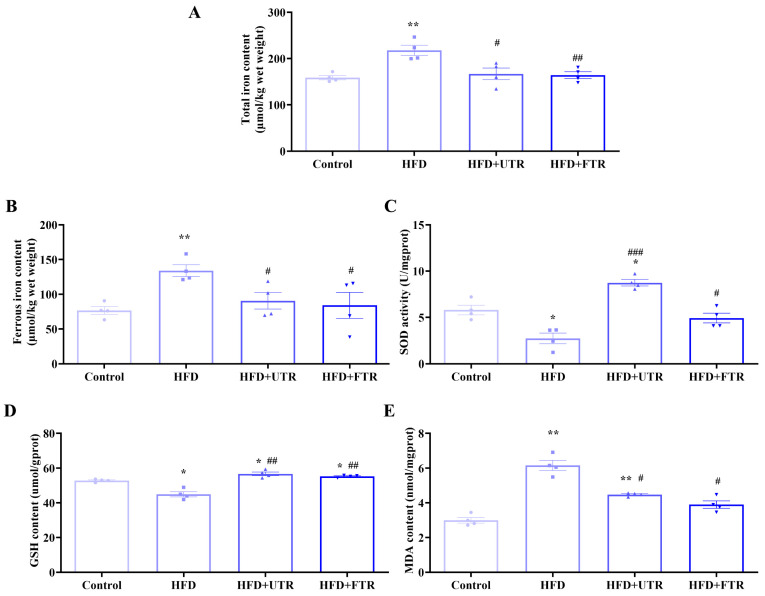
Exercise improves oxidative stress in the gastrocnemius muscle of sarcopenic obese mice induced by high-fat diet (HFD). (**A**) HFD induces an increase in the total iron content of the gastrocnemius muscle in mice, while exercise intervention reduces the total iron content of the gastrocnemius muscle in mice (*n* = 4); (**B**) HFD induces an increase in the divalent iron content of the gastrocnemius muscle in mice, while exercise intervention reduces the divalent iron content (*n* = 4); (**C**) HFD induces a decrease in the SOD enzyme activity of the gastrocnemius muscle in mice, while exercise intervention increases the SOD enzyme activity (*n* = 4); (**D**) HFD induces a decrease in the GSH content of the gastrocnemius muscle in mice, while exercise intervention increases the GSH content of the gastrocnemius muscle in mice (*n* = 4); (**E**) HFD induces an increase in MDA content in the gastrocnemius muscle of mice, while exercise intervention reduces MDA content (*n* = 4). UTR: uphill treadmill running, FTR: flat treadmill running; * compared with the control group, *p* < 0.05, ** compared with the control group, *p* < 0.01; # compared with the HFD group, *p* < 0.05; ## compared with the HFD group, *p* < 0.01, ### compared with the HFD group, *p* < 0.01.

To investigate whether ferroptosis is involved in HFD-induced sarcopenic mice, ferroptosis-related markers were assessed. HFD-induced sarcopenic mice exhibited significant skeletal muscle iron overload. The total iron and ferrous iron contents of the gastrocnemius muscle in the HFD group were significantly higher than those in the control group (Figure 6A,B). Consistent with the increased iron accumulation, HFD model mice showed significantly decreased SOD activity and GSH content, alongside elevated MDA levels in the gastrocnemius muscle (Figure 6C–E). These results indicate that ferroptosis is involved in HFD-induced sarcopenia in mice.

Flat treadmill running significantly reduced total and ferrous iron contents in the gastrocnemius muscle compared to the HFD group. Meanwhile, antioxidant capacity was significantly increased, as evidenced by increased antioxidant enzyme SOD activity and GSH content, while the lipid peroxidation marker MDA was significantly decreased. Uphill treadmill running exerted similar beneficial effects: muscle iron overload was alleviated, antioxidant capacity in the gastrocnemius muscle was enhanced, and MDA levels were reduced, effectively mitigating oxidative stress. These results demonstrate that both flat and uphill treadmill running can effectively reverse HFD-induced pathological changes in ferroptosis-related biochemical indicators in sarcopenic obesity.

**Figure 7:**
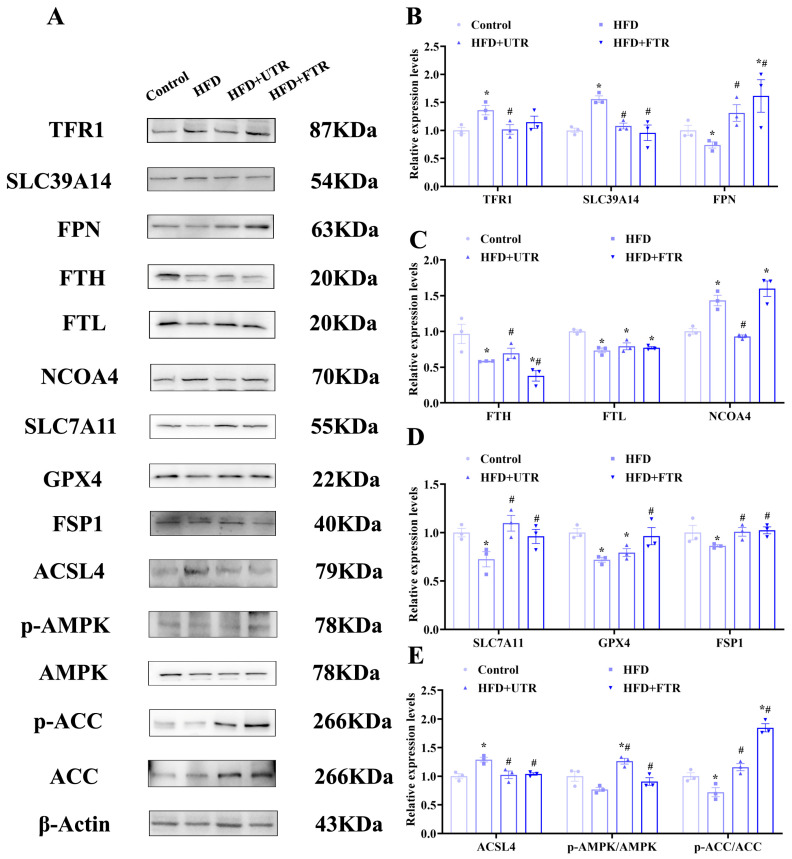
Exercise improves HFD-induced sarcopenic obesity in mice by inhibiting ferroptosis through activation of the adenosine monophosphate-activated protein kinase (AMPK)/Acetyl-CoA carboxylase pathway (ACC) pathway. (**A**) Expression of ferroptosis-related proteins and AMPK/ACC pathway proteins; (**B**) expression analysis of TFR1, SLC39A14, and FPN in the gastrocnemius muscle of mice in each group (*n* = 3); (**C**) expression analysis of FTH, FTL, and NCOA4 in the gastrocnemius muscle of mice in each group (*n* = 3); (**D**) expression analysis of SLC7A11, GPX4, and FSP1 in the gastrocnemius muscle of mice in each group (*n* = 3); (**E**) expression analysis of ACSL4, phosphorylated AMPK (*p*-AMPK), and phosphorylated ACC (*p*-ACC) in the gastrocnemius muscle of mice in each group (*n* = 3). * Compared with the control group, *p* < 0.05, # compared with the HFD group, *p* < 0.05.

Ferroptosis-related protein expression in the gastrocnemius muscle was also detected (Figure 7A). Compared with the control group, the HFD group exhibited significant upregulation of ferroptosis-promoting proteins in the gastrocnemius muscle, including TFR1, SLC39A14, NCOA4, and ACSL4. Conversely, expression of ferroptosis-inhibiting proteins, including FTL, FTH, SLC7A11, GPX4, and ferroptosis suppressor protein 1 (FSP1), was significantly decreased in the HFD group. These data further support ferroptosis as an important mechanism underlying HFD-induced sarcopenic obesity in mice (Figure 7B–E).

It is notable that both flat and uphill treadmill running interventions have shown significant regulatory effects on these proteins. Following exercise intervention, the expression of TFR1, SLC39A14, and NCOA4 in the gastrocnemius muscle decreased, while the expression of ferroportin (FPN) and FTL significantly increased. These changes suggest that exercise may ameliorate iron metabolism dysregulation by regulating iron transporters and iron storage proteins, thereby promoting the excretion and storage of iron. Additionally, exercise significantly decreased ACSL4 levels and upregulated SLC7A11, GPX4, and FSP1 expression in the gastrocnemius muscle. These findings indicate that exercise enhances muscle antioxidant defense capacity by upregulating antioxidant enzymes, reducing lipid peroxidation, alleviating oxidative stress, and ultimately inhibiting ferroptosis.

### 2.10. Exercise May Inhibit HFD-Induced Skeletal Muscle Ferroptosis via AMPK/ACC Pathway Activation

Adenosine monophosphate-activated protein kinase (AMPK) is a key regulator of cellular energy metabolism. Upon activation, AMPK inhibits lipid synthesis enzymes and related transcription factors while promoting fatty acid β-oxidation to generate cellular energy, thereby reducing lipid synthesis substrates and accumulation [47,48,49,50]. Activated AMPK phosphorylates and inhibits its downstream substrate acetyl-CoA carboxylase (ACC), suppressing fatty acid synthesis and reducing lipid peroxide accumulation to inhibit ferroptosis [47]. Compared with the control group, there were no significant differences in the total levels of AMPK and ACC in the gastrocnemius muscles of HFD-induced model mice. However, the levels of phosphorylated AMPK (p-AMPK) and phosphorylated ACC (p-ACC) were significantly decreased, indicating that HFD promotes lipid synthesis in gastrocnemius muscle tissues, which further confirms lipid deposition in these tissues (Figure 7A).

Both flat and uphill treadmill running interventions demonstrated significant regulatory effects by activating AMPK, as evidenced by a marked increase in p-AMPK levels. Concurrently, exercise enhanced p-ACC levels, effectively inhibiting fatty acid synthesis and reducing lipid peroxide accumulation (Figure 7E). These findings indicate that exercise activates the AMPK/ACC pathway, with AMPK-mediated ACC phosphorylation suppressing fatty acid synthesis and lipid peroxide accumulation, which may contribute to exercise-induced inhibition of skeletal muscle ferroptosis.

### 2.11. Activation of the AMPK/ACC Pathway Inhibits PA-Induced Ferroptosis of C2C12 Myoblasts

**Figure 8:**
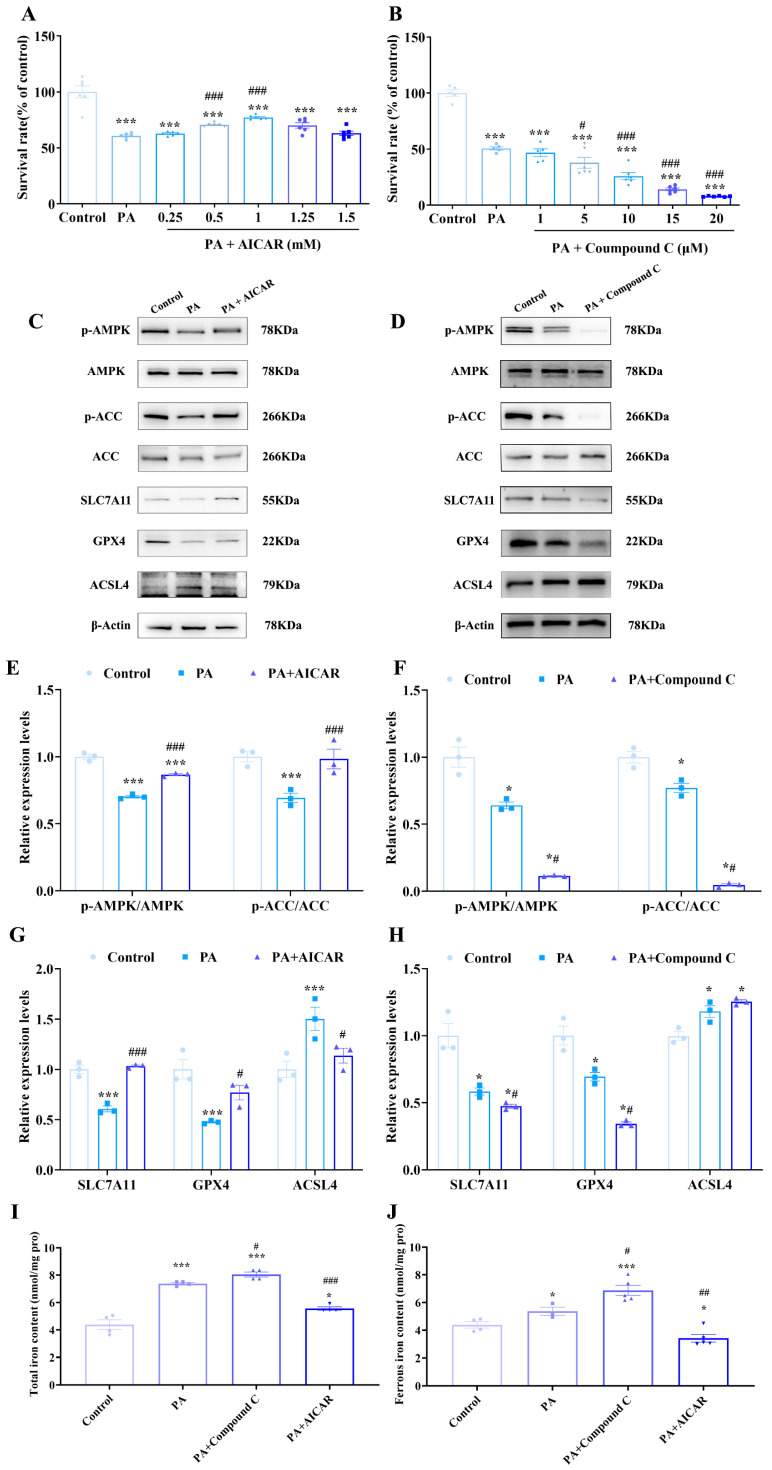
Activation of the AMPK/ACC pathway can inhibit PA-induced ferroptosis of C2C12 cells. (**A**) The effects of PA (500 μmol/L) and AMPK agonist 5-aminoimidazole-4-carboxamide 1-β-D-ribofuranoside (AICAR) on the proliferation of C2C12 cells; MTT assays were conducted at least three times, with representative results shown. (**B**) The effects of PA (500 μmol/L) and AMPK inhibitor Compound C on the proliferation of C2C12 cells; MTT assays were conducted at least three times, with representative results shown. (**C**) Expression of ferroptosis-related proteins and AMPK/ACC pathway proteins after PA (500 μmol/L) and AICAR treatment (1 mmol/L). (**D**) Expression of ferroptosis-related proteins and AMPK/ACC pathway proteins after PA (500 μmol/L) and Compound C treatment (10 μmol/L). (**E**) PA can inhibit the phosphorylation of AMPK and ACC in C2C12 cells, which can be reversed by AICAR (*n* = 3). (**F**) PA can inhibit the phosphorylation of AMPK and ACC in C2C12 cells, which can be aggravated by Compound C (*n* = 3). (**G**) Ferroptosis of C2C12 cells induced by PA can be reversed by AICAR (*n* = 3). (**H**) Ferroptosis of C2C12 cells induced by PA can be aggravated by Compound C (*n* = 3). (**I**) Detection of total iron content in cells of each group (*n* = 4). (**J**) Detection of ferrous iron content in cells of each group (n = 4). * Compared with the control group, *p* < 0.05, *** compared with the control group, *p* < 0.01; # compared with the PA group, *p* < 0.05; ## compared with the PA group, *p* < 0.01, ### compared with the PA group, *p* < 0.001.

To further explore the relationship between AMPK/ACC pathway activation and myocyte ferroptosis, PA-induced C2C12 cells were treated with the AMPK agonist 5-aminoimidazole-4-carboxamide 1-β-D-ribofuranoside (AICAR) and AMPK inhibitor Compound C. The results of the MTT assay showed that AICAR (0.25–1 mM) significantly reversed PA-induced inhibition of C2C12 cell proliferation (Figure 8A), and Compound C (5–20 μM) significantly exacerbated the proliferation inhibition of C2C12 cells induced by PA (Figure 8B), suggesting that activating AMPK may promote the proliferation of C2C12 cells by inhibiting PA-induced ferroptosis, while inhibiting AMPK may have the opposite effect [51]. Compared with the control group, there was no significant difference in the total levels of AMPK and ACC in C2C12 cells in the PA treatment group. However, p-AMPK and p-ACC levels were significantly decreased, indicating that PA induction promoted lipid synthesis in C2C12 cells. AICAR treatment significantly elevated p-AMPK and p-ACC levels in C2C12 cells, confirming AMPK/ACC pathway activation (Figure 8B,C). Conversely, Compound C treatment significantly reduced the levels of p-AMPK and p-ACC in C2C12 cells (Figure 8B,C).

Subsequently, ferroptosis-related protein expression was assessed (Figure 8D). AICAR upregulated the expression of key ferroptosis-inhibiting proteins SLC7A11 and GPX4 while downregulating the ferroptosis-promoting protein ACSL4. These changes collectively inhibited lipid peroxidation and ferroptosis in C2C12 cells. Conversely, Compound C exacerbated the reduction in SLC7A11 and GPX4 induced by PA and increased the expression of ACSL4, demonstrating that inhibiting the AMPK pathway can aggravate PA-induced ferroptosis. Compound C could intensify the iron accumulation in cells caused by PA, while AICAR could reverse this process. These findings support that activating the AMPK/ACC pathway can inhibit fatty acid synthesis, reduce the accumulation of lipid peroxides, and thus alleviate PA-induced ferroptosis in C2C12 cells.

## 3. Discussion

Sarcopenic obesity has emerged as a major public health challenge [10,52,53,54], which not only impairs physical functions, but also imposes substantial economic burdens on healthcare systems [55,56]. Our results highlight ferroptosis as a potential pathogenic driver in HFD-induced sarcopenic obesity, and exercise may exert protective effects by activating the AMPK/ACC pathway to inhibit ferroptosis, providing new insights and potential therapeutic strategies for the prevention and management of sarcopenic obesity.

The pathogenesis of sarcopenic obesity is multifactorial, involving the intricate interplay of hormonal dysregulation, chronic inflammation and cytokine imbalance, systemic lipid metabolism disorders, excessive oxidative stress and mitochondrial dysfunction, all of which synergistically contribute to the progressive loss of skeletal muscle mass and function, alongside aberrant adipose tissue accumulation [57,58,59,60]. Impaired insulin sensitivity, age-related sex hormone declines, and dysregulated adipokine secretion disrupt muscle anabolism and promote fat deposition [61,62]. Chronic low-grade inflammation, characterized by increased pro-inflammatory cytokines and decreased anti-inflammatory mediators, inhibits muscle protein synthesis and accelerates catabolism [58]. Excess circulating free fatty acids induce muscle lipotoxicity, disrupting insulin signaling and impairing muscle metabolic and contractile function [59,60]. Mitochondrial dysfunction, characterized by reduced biogenesis and impaired oxidative phosphorylation efficiency, not only limits ATP production but also promotes excessive rROS release; when coupled with compromised antioxidant defense, this induces oxidative damage to muscle macromolecules, disrupts anabolic signaling, accelerates muscle atrophy, and perpetuates a vicious cycle of muscle dysfunction and fat accumulation [57]. To clarify the pathogenic mechanism and the effect of exercise intervention, we first established an HFD-induced sarcopenic obesity model in mice. The results showed that HFD-fed mice exhibited increased body weight, elevated fat mass and fat ratio, reduced skeletal muscle mass and muscle index, and impaired motor function, indicating that HFD successfully induced sarcopenic obesity in mice (Figure 4). Further detection of inflammation factors revealed that compared with controls, the HFD group had significantly elevated serum pro-inflammatory interleukin-1β (IL-1β) and reduced anti-inflammatory interleukin-10 (IL-10). Although flat and uphill treadmill running showed trends of decreasing IL-1β and increasing IL-10, there were no statistically significant differences compared with the HFD group (Figure S2). For glucose metabolism, the HFD group exhibited higher fasting blood glucose (FBG), fasting insulin (FI), and oral glucose tolerance test (OGTT) glucose area under the curve (AUC) than controls, and neither exercise mode reversed these abnormalities (Figure S3). These results suggest that the effect of exercise on improving HFD-induced sarcopenic obesity may be independent of systemic inflammation regulation or glucose metabolism modulation, necessitating further exploration of other core pathogenic pathways.

Iron homeostasis is critical for maintaining cellular and organismal physiology [63], with iron overload promoting Fenton reaction and excessive free radical generation, which leads to ferroptosis and tissue damage [64]. Previous studies have implicated HFD-induced ferroptosis in cardiomyocytes and hepatocytes, contributing to the pathogenesis of non-alcoholic fatty liver disease and cardiac injury [65,66], offering potential insights into sarcopenic obesity. Studies have shown that skeletal muscle iron overload exists in patients with sarcopenic obesity and model animals [67], with such overload potentially catalyzing ROS production, promoting lipid peroxidation and ferroptosis, and contributing to muscle mass loss and functional decline. Consistent with these studies, HFD-fed mice in this study exhibited significantly increased total iron and ferrous iron concentrations in skeletal muscle, suggesting decreased stable iron storage and increased labile free iron (Figure 6). Similar trends of increased total iron and ferrous iron were observed in C2C12 cells treated with PA (Figure 2). A recent study by Chang et al. demonstrated that sodium palmitate could enhance the FerroOrange signal in pancreatic β-cells, which is consistent with our findings regarding the content of ferrous ions [68]. These data confirm the existence of abnormal iron regulation in sarcopenic obesity, suggesting that skeletal muscle iron metabolism disorders may be an important initiating factor in the pathophysiological process of sarcopenic obesity. The membrane iron transporter SLC39A14 mediates non-transferrin binding iron absorption and leads to the accumulation of free iron in skeletal muscle [69]. Conversely, FPN is the primary mammalian iron exporter [70] and has been reported to be downregulated in obese patients and models alongside upregulated SLC39A14, and these changes are closely related to iron overload [71,72]. Here, HFD-fed mice and PA-treated C2C12 cells exhibited upregulation of both TFR1 and SLC39A14 as well as downregulation of FPN (Figure 3 and Figure 7). This dual upregulation of iron uptake transporters, combined with impaired iron export, suggests a multi-faceted mechanism underlying skeletal muscle iron overload in sarcopenic obesity in which enhanced iron influx via both transferrin-dependent and transferrin-independent pathways, coupled with reduced iron efflux, synergistically exacerbates labile iron accumulation and subsequent ferroptosis. Notably, exercise intervention reversed these perturbations by downregulating TFR1 and SLC39A14 expression, increasing FPN levels, and ultimately restoring iron homeostasis in skeletal muscle. Combined with exercise-related changes in FTL and FTH expression (Figure 3 and Figure 7), these findings suggest that exercise may improve iron metabolism by reducing non-heme iron uptake, enhancing iron export, and promoting iron storage and ferritin synthesis, potentially alleviating obesity-induced sarcopenia by reducing free iron accumulation.

Lipid metabolic dysregulation in sarcopenic obesity may further exacerbate ferroptosis. HFD-induced adipose tissue dysfunction leads to the release of excessive free fatty acids, which then accumulate abnormally in the muscles. Polyunsaturated fatty acids (PUFAs), in particular, are highly susceptible to peroxidation, providing substrates for ferroptosis. The lipid metabolism enzyme ACSL4 catalyzes the biochemical reaction between free arachidonic acid and coenzyme A and promotes its esterification into phospholipids, which is necessary for the formation of phospholipid peroxides (PL-OOH) [63]. Then, the arachidonate lipoxygenase (ALOX) family (mainly ALOX12) mediates the peroxidation of PUFAs, leading to the release of a large amount of lipid ROS. Amino acid metabolism is also closely related to ferroptosis: cysteine uptake via the system XC^−^ transporter (with SLC7A11 as a specific subunit) supports GSH synthesis and antioxidant activity [26]. Obese individuals with sarcopenia have decreased antioxidant capacity, including lower GPX4 and GSH levels, which may fail to inhibit lipid peroxidation, further promoting ferroptosis. Consistent with these reports, the present study found that HFD increased skeletal muscle MDA while reducing SOD activity and GSH levels (Figure 6), and PA-treated C2C12 cells exhibited ROS accumulation and deprivation of GSH and SOD, which were partially reversed by the ferroptosis inhibitor Fer-1 (Figure 2). These findings are consistent with the possibility that ferroptosis may contribute specifically to sarcopenic obesity. Exercise intervention effectively increased GSH content, upregulated SLC7A11 and GPX4, reduced ACSL4 expression and MDA levels, and enhanced SOD activity (Figure 6 and Figure 7), which were consistent with the possibility that exercise may inhibit HFD-induced ferroptosis by enhancing antioxidant capacity and inhibiting lipid peroxidation, thereby improving sarcopenic obesity.

AMPK is an important sensor for energy metabolism and is crucial for sensing and regulating intracellular energy metabolism. Its main functions include regulating lipid metabolism and inhibiting protein synthesis [73,74,75]. Under energy stress conditions, such as aerobic exercise, increased ATP consumption may elevate AMPK expression and phosphorylation to maintain cellular energy homeostasis [76,77,78]. Based on the energy regulatory properties of AMPK, we hypothesized that it may be involved in the regulation of ferroptosis, so we further explored the activation status of AMPK in muscle cells under different energy and nutritional conditions. The results showed that PA treatment significantly downregulated p-AMPK levels, while sugar-free medium treatment (simulating moderate energy deprivation) promoted the phosphorylation activation of AMPK and was associated with an increased expression level of FPN (Figure S4).

**Figure 9:**
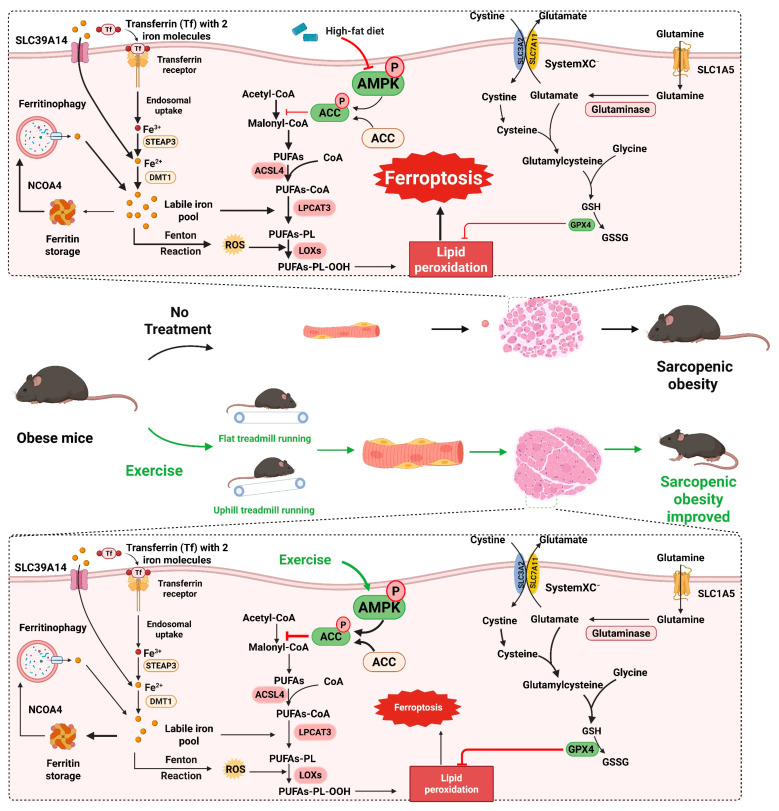
The mechanism hypothesis diagram of this research. High-fat diet (HFD) can inhibit the phosphorylation activation of the AMPK/ACC pathway, suppress the antioxidant capacity of cells, promote the synthesis of fatty acids and lipid peroxidation, thereby inducing ferroptosis of muscle cells and causing sarcopenic obesity in mice. Exercise intervention, including resistance exercise and aerobic exercise, can activate the AMPK/ACC pathway, enhance the antioxidant capacity of cells, inhibit the synthesis of fatty acids and lipid peroxidation, thereby suppressing ferroptosis of muscle cells and significantly improving sarcopenic obesity in mice.

Growing evidence suggests AMPK may modulate ferroptosis. Lee and his colleagues found that inducing or simulating energy stress inhibited ferroptosis, with cancer cells exhibiting high AMPK activity showing resistance to ferroptosis, while AMPK inactivation increased ferroptosis sensitivity, and the regulation of ferroptosis by AMPK was related to AMPK-mediated ACC phosphorylation and polyunsaturated fatty acid biosynthesis [38]. Activation of the AMPK/ACC pathway may inhibit fatty acid synthesis, reducing the accumulation of lipid peroxides and thereby suppressing ferroptosis [79,80]. Additionally, the activated AMPK/ACC pathway has been reported to upregulate GPX4, downregulate ACSL4, and alleviate high glucose-induced hepatocyte ferroptosis, with AMPK inhibitors reversing these effects, which suggests that the AMPK/ACC pathway may mediate ferroptosis inhibition [81]. In the present study, HFD feeding significantly reduced the expression levels of p-AMPK and p-ACC in the gastrocnemius muscle (Figure 7), which was consistent with the results of Oil red O staining and the Western blotting detection of ferroptosis-related proteins, suggesting that inhibition of the AMPK/ACC pathway may exacerbate ferroptosis by promoting lipid synthesis (including unsaturated fatty acids). Exercise intervention significantly upregulated the expression of p-AMPK and p-ACC in the gastrocnemius muscle, coinciding with reduced ferroptosis (Figure 7) and supporting the possibility that exercise may inhibit skeletal muscle ferroptosis via AMPK/ACC pathway activation, thereby improving sarcopenic obesity. Cell experiments further confirmed that AMPK agonist AICAR treatment was associated with AMPK activation, upregulation of GPX4, and downregulation of ACSL4, while AMPK inhibitor Compound C could exacerbate ferroptosis in cells by inhibiting the activation of the AMPK pathway (Figure 8), which is consistent with the hypothesis that activating the AMPK/ACC pathway may reverse high-fat-induced ferroptosis. These results collectively indicate that exercise-induced ferroptosis inhibition may partially depend on AMPK activation, with AMPK-mediated ferroptosis signaling potentially playing a role in exercise-related improvements in HFD-induced sarcopenic obesity (Figure 9).

Despite the novel insights gained from the present study, several limitations should be acknowledged. First, direct measurement of the labile iron pool (LIP) using specific probes was not performed, which limits the direct confirmation of iron-dependent ferroptosis in skeletal muscle cells. Future studies will employ FerroOrange or FeRhoNox-1 to validate HFD/PA-induced LIP elevation, which will further solidify the causal link between iron overload and ferroptosis. Second, the causal link between AMPK activation and ferroptosis inhibition lacks genetic validation. Genetic models, including AMPK knockdown/knockout mice or C2C12 cells, will be used in subsequent work to clarify whether AMPK deficiency abrogates the protective effects of exercise. Third, this study focused primarily on the gastrocnemius muscle, a mixed fiber-type muscle, while the slow-twitch soleus muscle, which is rich in mitochondria and more susceptible to iron-mediated oxidative stress, may exhibit more pronounced ferroptosis. Systematic comparisons of ferroptosis in different muscle fiber types are needed to confirm fiber-specific mechanisms. These limitations provide important directions for refining the mechanistic understanding of exercise-mediated protection against sarcopenic obesity.

In summary, the present study provides evidence supporting ferroptosis as a potential pathogenic mechanism in HFD-induced sarcopenic obesity and points to the AMPK/ACC pathway as a plausible mediator of exercise-induced ferroptosis inhibition. These findings provide a preliminary theoretical basis for the mechanism by which exercise improves sarcopenic obesity and offer experimental support for the development of intervention strategies targeting iron-lipid metabolism crosstalk and the AMPK/ACC signaling pathway.

## 4. Materials and Methods

### 4.1. Cell Culture and Myogenic Differentiation

Murine myoblast C2C12 cells (obtained from Wuhan Servicebio Technology Co., Ltd., Wuhan, China) were cultured in high-glucose Dulbecco’s Modified Eagle Medium (DMEM) plus 10% fetal bovine serum following standard cell culture protocols. To prepare a 100 mmol/L palmitate stock, the palmitic acid was complexed to 10% fatty-acid-free bovine serum albumin (BSA) at 37 °C with constant agitation. In the palmitic acid toxicity experiment, C2C12 cells were treated with serial palmitic acid doses comprising 1% fat-free BSA for 24 h. Other cell experiments employed 500 μmol/L palmitic acid (which contained 1% fat-free BSA) with or without additional compounds, while controls received vehicle (1% fatty-acid-free BSA) alone [82]. To trigger differentiation, cells were shifted to high-glucose DMEM containing 2% horse serum once confluency reached approximately 80% [83]. Differentiation was monitored through morphological changes. Over six days, mononucleated spindle cells elongated, fused into multinucleated tubes, and aligned in parallel arrays, confirming successful myotube formation [84,85].

### 4.2. MTT Assay

Briefly, C2C12 cells were plated at 5 × 10^3^ cells per well of 96-well plates and allowed to attach overnight. Following treatment with different experimental conditions as designed, 10 μL of MTT solution was added and incubation continued for 4 h at 37 °C. After incubation, the supernatant was carefully removed, and the violet formazan precipitate was solubilized with 150 μL dimethyl sulfoxide (DMSO). Absorbance of each well was read at 570 nm on a FlexA-200HT microplate spectrophotometer (Hangzhou Allsheng Instruments Co., Ltd., Hangzhou, China). Cell viability was expressed as the ratio of treated to control optical densities multiplied by 100%. Each MTT assay was conducted at least three times.

### 4.3. Iron Staining

Sterilized coverslips were placed in 24-well plates and seeded with an appropriate volume of cell suspension to ensure complete coverage [86]. After treatment, culture medium was removed and cell-covered coverslips were gently rinsed three times with pre-warmed phosphate-buffered saline (PBS), then fixed with ice-cold methanol (4 °C, 15 min). After fixation, coverslips were washed three times with PBS to remove residual fixative. Fixed coverslips were carefully transferred to a staining rack in a staining dish and incubated for 30 min at room temperature in a freshly prepared solution containing equal volumes of 4% hydrochloric acid and 4% potassium ferrocyanide. Endogenous peroxidase was blocked with 1% hydrogen peroxide in methanol (20 min), and samples were rinsed with PBS three times. Subsequently, coverslips were incubated with 3,3-diaminobenzidine (DAB) until brown granules became visible. The staining reaction was terminated by rinsing with distilled water, and coverslips were mounted with neutral gum.

### 4.4. Measurement of Reactive Oxygen Species (ROS)

ROS levels were measured using the fluorescent probe 2′,7′-dichlorodihydrofluorescein diacetate (DCFH-DA, Wuhan Servicebio Technology Co., Ltd., Wuhan, China). C2C12 cells seeded on sterilized coverslips were rinsed twice with warm PBS after treatments and incubated with 100 μmol/L DCFH-DA for 1 h. Following the incubation, excess DCFH-DA was removed by two additional PBS washes. Fluorescence microscopy (TS2-FL, Nikon Corporation, Tokyo, Japan) was used to visualize ROS-dependent fluorescence. Representative images were photographed, and the relative fluorescence intensity was calculated with ImageJ 1.53c.

### 4.5. Transmission Electron Microscopy (TEM) of Myoblasts

C2C12 myoblasts were fixed overnight at 4 °C in 2.5% glutaraldehyde, post-fixed with 1% osmium tetroxide, and then dehydrated through graded ethanol and acetone. After infiltration in epoxy resin and polymerization at 60 °C, 70 nm ultrathin sections were cut on a Leica UC7 ultramicrotome (Leitz Camera, Wetzlar, Germany) [46]. The stained sections were imaged in a Hitachi HT-7800 transmission electron microscope (Hitachi Limited, Hong Kong, China) to observe the ultrastructural features of the myoblasts.

### 4.6. Animal Experiments and Exercise Interventions

C57BL/6 mice (male, 22 ± 2 g, 8 weeks old) were provided by Jiangsu Huachuang Xinnuo Pharmaceutical Technology Co., Ltd. (animal qualification certificate number: 202356879, Nanjing, China), and were kept at 22 ± 2 °C on a 12 h light/dark cycle with free access to water and standard chow. After 7 d acclimation, animals were randomly assigned to four groups: control group (CON), high-fat diet group (HFD), HFD + flat treadmill running group (HFD + FTR), and HFD + uphill treadmill running group (HFD + UTR). Animals in the HFD, HFD + FTR and HFD + UTR groups were fed a high-fat diet (XTHF60, XIETONG.ORGANISM), while mice in the CON group received a control chow diet (XTCON50J, XIETONG.ORGANISM).

**Figure 10:**
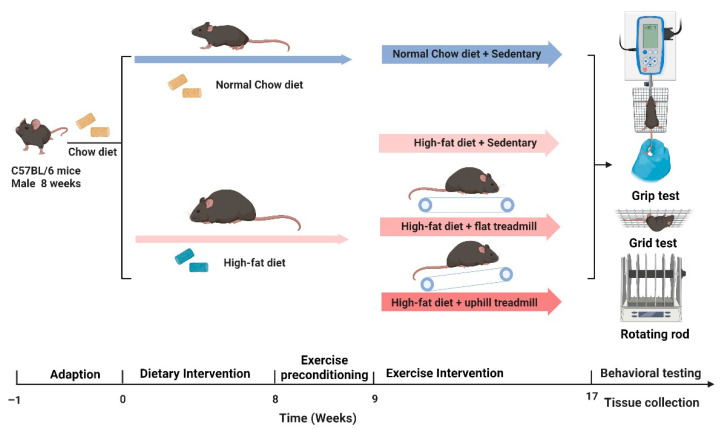
Schematic of the animal study design and exercise intervention protocol.

A small animal treadmill (XR-PT-10B, Shanghai Xinruan Information Technology Co., Ltd., Shanghai, China) was used for exercise interventions. After eight weeks of dietary treatment, mice in the HFD + FTR group underwent a standardized aerobic exercise protocol: five days of adaptation at 6 m/min (0% slope), increasing from 10 to 50 min. Subsequently, mice ran 60 min per day (0% slope, 5 min warm-up at 6 m/min, 50 min at 9 m/min, and 5 min cool-down at 6 m/min), five days/week for eight weeks. Mice in the HFD + UTR group followed a similar protocol with a 15% slope. In contrast, mice in the HFD group were placed on stationary treadmills for eight weeks to provide a similar environmental stress. Diets were maintained throughout the intervention period. The animal experiment timeline is outlined in Figure 10. Procedures were approved by the Jianghan University Animal Ethics Committee.

### 4.7. Body Composition Analysis

After the eight-week intervention, lean and fat mass were non-invasively quantified in conscious mice by nuclear magnetic resonance using a Minispec LF-50 analyzer (Bruker, Berlin, Germany).

### 4.8. Behavioral Assessments

Grip strength test: Fore- and hind-limb grip force were evaluated with a grip strength meter (XR501, Shanghai Xinruan Information Technology Co., Ltd., Shanghai, China). Each mouse was held above the grid and pulled gently by the tail until release; the peak force from three consecutive trials was averaged [87].

Hanging grid test: Animals were placed individually on a 2 mm wire grid positioned 50 cm above a padded surface. The grid was then inverted, and the latency to fall was measured [88]. Each mouse completed three trials with a 30 min interval, and the data from all three trials were averaged.

Rotating rod test: Motor function was assessed using a rotarod apparatus (XR-YLS-10B, Shanghai Xinruan Information Technology Co., Ltd.) with two protocols: fixed-speed and accelerated rotation. For the fixed-speed test, mice were placed on a rod rotating at 15 r/min with three trials separated by 30 min rest periods. For the accelerated test, mice were placed on a rod that accelerated smoothly from 5 r/min to 40 r/min at a uniform acceleration rate in 5 min. In both protocols, latency to fall (time spent on the rod before falling) was recorded [89].

### 4.9. Muscle Weight Measurements

Upon completion of the study, mice were euthanized, and the soleus muscles, gastrocnemius, and tibialis anterior were dissected, blotted free of blood, and weighed immediately on an analytical balance to obtain wet mass.

### 4.10. Histological Staining

HE Staining: After isoflurane anesthesia, mice underwent cardiac perfusion with paraformaldehyde. Gastrocnemius, tibialis anterior, and soleus muscle samples were immediately dissected and immersed in paraformaldehyde for fixation. After fixation, samples were dehydrated through graded ethanol solutions and xylene, then infiltrated with molten paraffin wax. Serial 5 µm sections (Leica Microsystems AG, Wetzlar, Germany) were de-waxed, rehydrated, and stained with Harris haematoxylin (5–8 min), followed by rinsing in running tap water to blue the nuclei. Then, sections were differentiated in 1% acid alcohol for 3–5 s, and re-blued in running tap water. After that, sections were stained with eosin Y solution for 2~3 min. Stained sections were dehydrated and finally mounted with coverslips using a synthetic resin-based mounting medium [90].

Oil Red O Staining: Fixed gastrocnemius samples were dehydrated with gradient sucrose solutions. Additionally, 10 µm thick frozen slices were obtained using a cryostat (CRYOSTAR NX50, Thermo Fisher Scientific, Waltham, MA, USA) and immediately mounted on gelatin-coated slides. Slides were post-fixed for 5~10 min in 4% paraformaldehyde, rinsed in 60% isopropanol, and incubated for 10–15 min in freshly filtered Oil Red O working solution. After a brief 60% isopropanol rinse, nuclei were counter-stained with haematoxylin and sections were coverslipped with aqueous mountant [91].

Digital micrographs were acquired on a PanoBrain analyzer (Tinyphoton (Wuhan) Technology Co., Ltd., Wuhan, China). Mean fiber cross-sectional area and lipid-droplet density were quantified in a blinded manner using ImageJ 1.53c.

### 4.11. Biochemical Assays in Cells and Animal Tissues

GSH, SOD, and MDA Assays: Cell lysates and tissue homogenates were prepared for biochemical analyses. SOD activities, GSH, and MDA levels were determined with commercial kits. GSH and SOD detection kits were obtained from Wuhan Servicebio Technology Co., Ltd., Wuhan, China and the MDA detection kit was purchased from Nanjing Jiancheng Bioengineering Research Institute. These assays evaluated oxidative stress status in cells and tissues. SOD activity was measured as U/mg protein, GSH content as μmol/mg protein, and MDA levels as nmol/mg protein.

Total Iron and Ferrous Iron Assays: Total and ferrous iron were quantified using specific colorimetric assays (Shanghai Yamay Biomedical Technology Co., Ltd., Shanghai, China), and values are reported as nmol/10^6^ cells or μmol/mg tissue weight.

### 4.12. Immunoblotting

Total protein was extracted with ice-cold radioimmunoprecipitation assay (RIPA) plus protease/phosphatase inhibitors, and concentrations were determined with a bicinchoninic acid (BCA) assay. Equal amounts of protein were separated by sodium dodecyl sulfate-polyacrylamide gel electrophoresis (SDS-PAGE) and electro-transferred to polyvinylidene fluoride (PVDF) membranes. After 1 h blocking in 5% non-fat milk, membranes were incubated overnight at 4 °C with the following primary antibodies (all from Absin, 1:1000 unless stated): anti-TFR1, anti-SLC39A14, anti-FPN, anti-FTH, anti-FTL, anti-NCOA4, anti-SLC7A11, anti-GPX4, anti-FSP1, and anti-ACSL4; β-actin (1:2000, servicebio) served as the loading control. Following three TBST washes, horseradish peroxidase-conjugated secondary antibodies were applied for 1 h at room temperature. Protein bands were visualized using chemiluminescence reagents, and the intensity of the bands was quantified using Image J software. The relative expression levels of phosphorylated proteins (p-AMPK and p-ACC) were normalized to their corresponding total protein levels (AMPK and ACC), while the expression levels of all other proteins were normalized to housekeeping protein β-actin.

### 4.13. Statistical Analysis

Data were expressed as the mean ± standard error of the mean (SEM). All comparisons were performed with SPSS 23.0. Protein-expression datasets that deviated from normality or homoscedasticity were analyzed with the non-parametric Kruskal–Wallis test; other data were examined by one-way analysis of variance (ANOVA) followed by Tukey’s honest significant difference (HSD) post hoc test. *p*-value < 0.05 was taken as the threshold for statistical significance.

## 5. Conclusions

The incidence of sarcopenic obesity has increased sharply in recent years, with its pathogenesis incompletely understood and effective interventions urgently needed. This study provides evidence supporting that ferroptosis plays a critical role in HFD-induced sarcopenic obesity and suggests potential mechanisms by which exercise may inhibit ferroptosis, possibly through AMPK/ACC pathway activation. HFD induced skeletal muscle iron metabolism imbalance and exacerbated lipid peroxidation via dysregulated lipid metabolism, which may collectively contribute to myocyte ferroptosis and lead to muscle mass loss and functional decline. Exercise intervention reversed these changes, with activation of the AMPK/ACC pathway coinciding with reduced fatty acid synthesis, improved iron metabolism regulation, and enhanced antioxidant capacity. These effects synergistically inhibit ferroptosis and improve the pathological changes in skeletal muscle induced by HFD. Future studies may further explore combined exercise strategies targeting the AMPK/ACC pathway, potentially offering novel insights for the precise prevention and treatment of sarcopenic obesity. These findings not only yield novel mechanistic insights into exercise-mediated skeletal muscle protection but also offer a theoretical basis for developing clinical interventions. Such precision strategies hold substantial clinical promise for preventing, mitigating, and managing obesity-associated sarcopenia, ultimately improving patients’ physical function and enhancing long-term quality of life.

## Data Availability

The original contributions presented in this study are included in the article/Supplementary Materials. Further inquiries can be directed to the corresponding authors.

## Supplementary Materials

The following supporting information can be downloaded at: https://www.mdpi.com/article/10.3390/ijms27031187/s1.

## Abbreviations

3-MA	3-methyladenine
ACC	Acetyl-CoA carboxylase
ACSL4	Acyl-CoA synthase long-chain family member 4
AICAR	5-aminoimidazole-4-carboxamide 1-β-D-ribofuranoside
ALOX	Arachidonate lipoxygenase
ALOX12	Arachidonate-12-lipoxygenase
AMPK	Adenosine monophosphate-activated protein kinase
ANOVA	Analysis of variance
AUC	Area under the curve
BCA	Bicinchoninic acid
BSA	Bovine serum albumin
CoA	Coenzyme A
CSA	Cross-sectional area
DALYs	Disability-adjusted life years
DAB	3,3-diaminobenzidine
DCFH-DA	2′,7′-dichlorodihydrofluorescein diacetate
DMEM	Dulbecco’s modified eagle medium
DMSO	Dimethyl sulfoxide
DMT1	Divalent metal transporter 1
FBS	Fetal bovine serum
FBG	Fasting blood glucose
Fer-1	Ferrostatin-1
FI	Fasting insulin
FPN	Ferroportin
FTH	Ferritin heavy chain
FTL	Ferritin light chain
FTR	Flat treadmill running
FSP1	Ferroptosis suppressor protein 1
GPX4	Glutathione peroxidase 4
GSH	Glutathione
GSSG	Oxidized glutathione disulfide
HE	Hematoxylin-eosin
HFD	High-fat diet
HSD	Honest significant difference
LOXs	lipoxygenases
LPCAT3	Lysophosphatidylcholine acyltransferase 3
MDA	Malondialdehyde
MTT	3-(4,5-dimethylthiazol-2-yl)-2,5-diphenyltetrazolium bromide
NCOA4	Nuclear receptor coactivator 4
OGTT	Oral glucose tolerance test
PA	Palmitic acid
p-ACC	Phosphorylated ACC
p-AMPK	Phosphorylated AMPK
PBS	Phosphate-buffered saline
PL	Phospholipid
PL-OOH	Phospholipid peroxides
PUFAs	Polyunsaturated fatty acids
PVDF	Polyvinylidene fluoride
RIPA	Radioimmunoprecipitation assay
ROS	Reactive oxygen species
SDS-PAGE	Sodium dodecyl sulfate-polyacrylamide gel electrophoresis
SEM	Standard error of the mean
SLC1A5	Solute carrier family 1 member 5
SLC7A11	Solute carrier family 7 member 11
SLC39A14	Solute carrier family 39 (zinc transporter), member 14
SOD	Superoxide dismutase
STEAP3	Six-transmembrane epithelial antigen of prostate 3
TEM	Transmission electron microscope
TF	Transferrin
TFR1	Transferrin receptor 1
UTR	Uphill treadmill running
