# Supplementary material for: Exercise Improves Sarcopenic Obesity Through Inhibition of Ferroptosis and Activation of the AMPK/ACC Pathway
Source: Int J Mol Sci. 2026 Jan 24;27(3):1187. doi: 10.3390/ijms27031187 (PMC12897762; doi:10.3390/ijms27031187)
Supplement: Supplementary file 1 [file ijms-27-01187-s001.zip › Figure legends of supplementary figures.pdf]

Figure legend

Figure S1 The effect of ferroptosis inhibitor ferrostatin-1 (Fer-1) on the proliferation of C2C12 cells. A. Fer-1 had no significant effect on the proliferation of C2C12 cells, MTT (3-(4,5-dimethylthiazol-2-yl)-2,5-diphenyltetrazolium bromide) assay was conducted at least three times, with representative results shown; B. Palmitic acid (PA, 500 μmol/L) can inhibit the proliferation of C2C12 cells, which can be reversed by Fer-1 (10 μmol/L), and treatment with Fer-1 alone has no significant effect on cell proliferation and cell viability. MTT assay were conducted at least three times, with representative results shown. *Compared with the control group, $p < 0.05$. ***compared with the control group, $p < 0.001$; ###compared with the PA group, $p < 0.001$.

Figure S2 The effect of exercise on the inflammatory response caused by a high-fat diet. A. Detection of serum interleukin-1β (IL-1β) content of each group; B. Detection of serum interleukin-10 (IL-10) content of each group. *Compared with the control group, $p < 0.05$.

Figure S3 The effect of exercise on glucose metabolism caused by a high-fat diet (HFD). A. Detection of fasting blood glucose content in serum of each group; B. Detection of fasting insulin content in serum of each group. C. Oral glucose tolerance test for each group; D. Analysis of the area under the curve of blood glucose in oral glucose tolerance test. UTR: uphill treadmill running, FTR: flat treadmill running. **Compared with the control group, p < 0.01.

Figure S4 The effect of high-fat and energy deprivation on C2C12 cells. A. Palmitic acid (PA) and sugar-free (simulating moderate energy deprivation) treatment inhibits the proliferation of C2C12 cells, MTT assay were conducted at least three times, with representative results shown; B. Expression of phosphorylated adenosine monophosphate-activated protein kinase (*p*-AMPK) and ferroportin (FPN); C. Expression analysis of *p*-AMPK in C2C12 cells (n=3); D. Expression analysis of FPN

in C2C12 cells (n=3). *Compared with the control group, $p < 0.05$, ***compared with the control group, $p < 0.001$, # compared with the PA group, $p < 0.05$.
