# Supplementary figures and images for: Exercise Improves Sarcopenic Obesity Through Inhibition of Ferroptosis and Activation of the AMPK/ACC Pathway
Source: Int J Mol Sci. 2026 Jan 24;27(3):1187. doi: 10.3390/ijms27031187 (PMC12897762; doi:10.3390/ijms27031187)

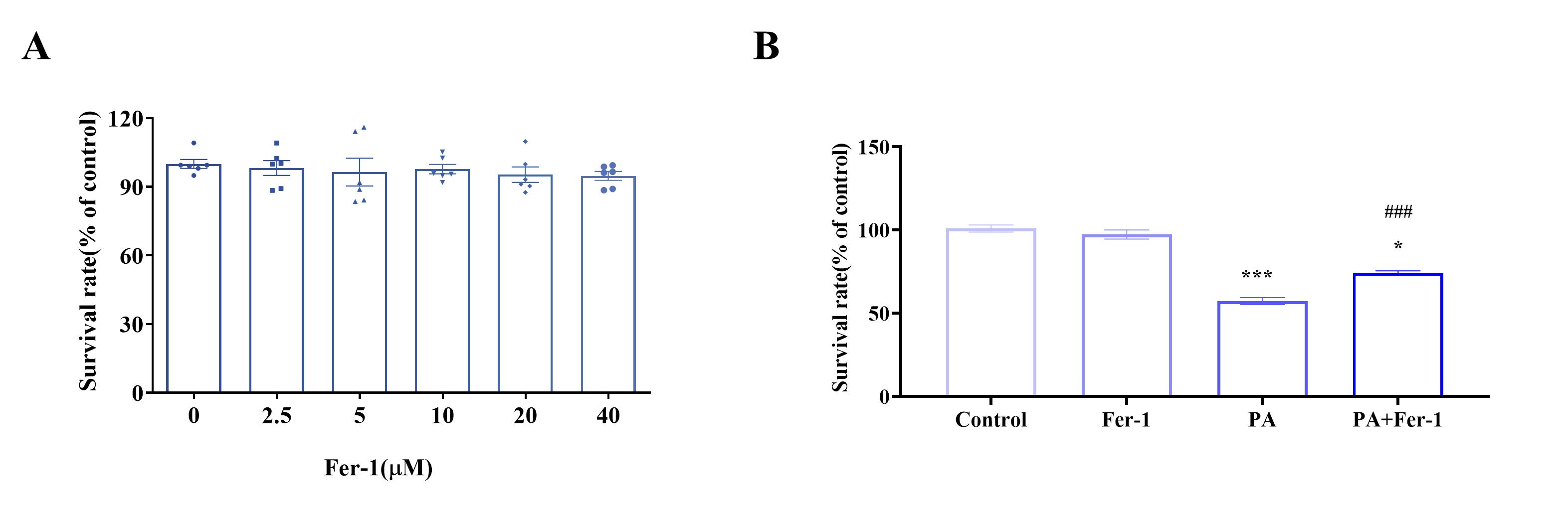

Supplement: Supplementary file 1 [file ijms-27-01187-s001.zip › S1.tif]

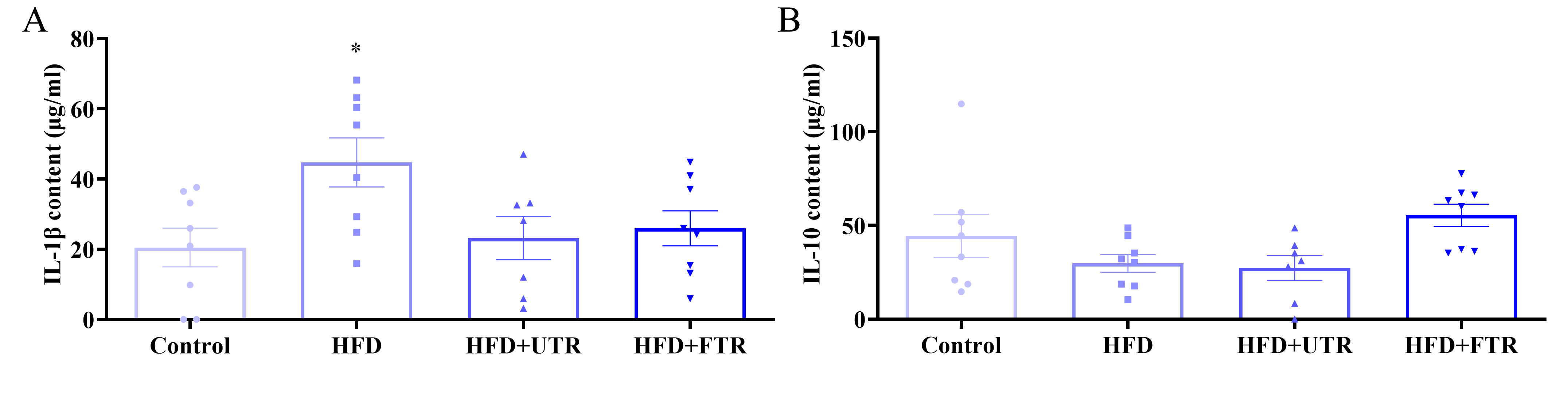

Supplement: Supplementary file 1 [file ijms-27-01187-s001.zip › S2.tif]

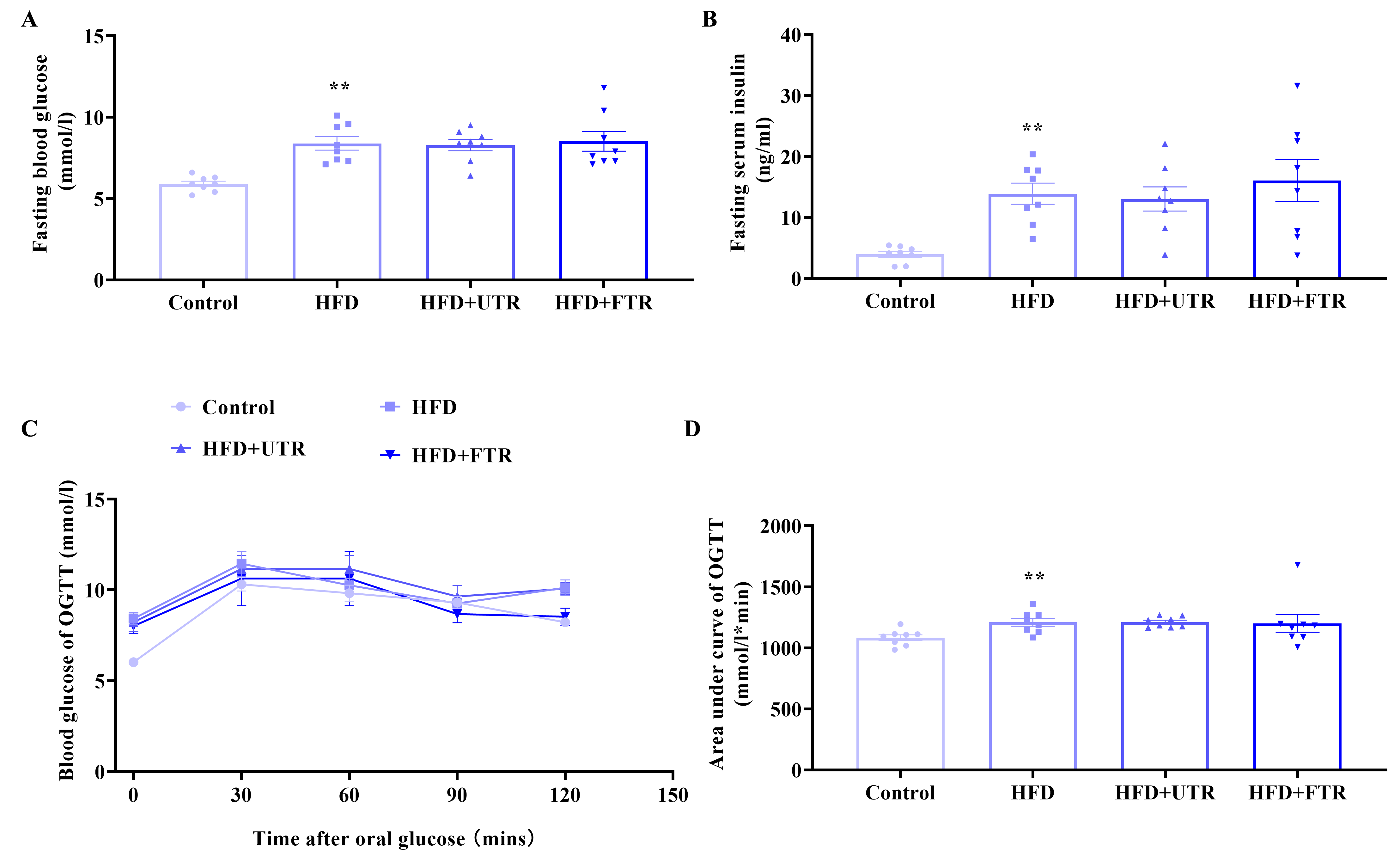

Supplement: Supplementary file 1 [file ijms-27-01187-s001.zip › S3.tif]

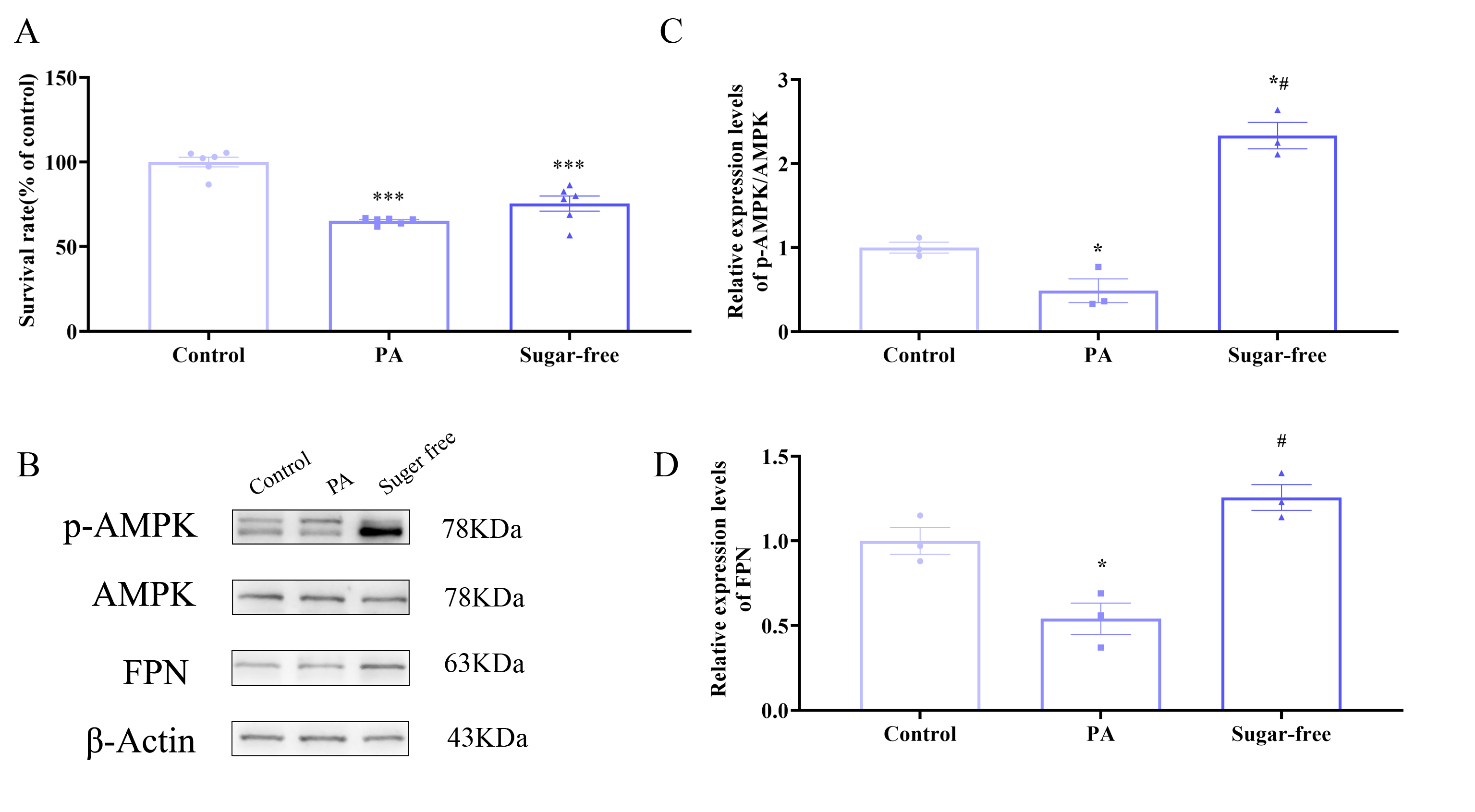

Supplement: Supplementary file 1 [file ijms-27-01187-s001.zip › S4.tif]
